# Delayed academic consolidation and active aging in European academia: a collaborative autoethnography of migrant women

**DOI:** 10.3389/fsoc.2026.1896968

**Published:** 2026-08-26

**Authors:** Mihaela Vancea, Noemí Duran-Salvadó, Valeria De Ormaechea-Otarola, Carolina Quirós-Domínguez

**Affiliations:** 1 Department of Research Methods and Diagnosis in Education, Universitat de Barcelona, Barcelona, Spain; 2 Department of Theory and History of Education, Universitat de Barcelona, Barcelona, Spain

**Keywords:** active aging, collaborative autoethnography, delayed academic consolidation, intersectionality, migrant women academics, recognition theory

## Abstract

**Introduction:**

Migrant women's opportunities for active aging, understood through the World Health Organization framework of health, participation, and security, are shaped by cumulative inequalities across the life course. In European higher education, migrant women academics often experience delayed academic consolidation through trajectories marked by precarious employment, disrupted institutional and relational ties, constrained recognition, and normative expectations that privilege linear and uninterrupted careers. This article examines how and why migrant women academics experience delayed academic consolidation and explores its implications for active aging.

**Methods:**

Grounded in collaborative autoethnography and informed by feminist intersectional perspectives, the study draws on first-person narratives and collective dialogic interpretation.

**Results:**

The analysis identifies four cross-cutting experiential dimensions: uprootedness and relational fragility; imposture; health and wellbeing; and modes of enacting the profession. These dimensions reveal how misrecognition, precarity, temporal dislocation, and work intensification shape academic life over time. The findings show that delayed academic consolidation is not only a career-related phenomenon, but also a life-course process with consequences for future health, participation, and security.

**Discussion:**

By bringing feminist intersectional perspectives, recognition theory, and the Active Aging framework into dialogue, the article conceptualizes delayed academic consolidation as a prolonged, non-linear, and structurally conditioned process through which academic recognition, participation, and stability are deferred as a result of intersecting inequalities. Methodologically, the study demonstrates the value of collaborative autoethnography for making visible embodied and situated forms of academic inequality that are often obscured in dominant accounts of academic work.

## Introduction

***Scene 1 (Author A): traces of unease***

*Today my daughter came over. A recent death, along with the formalities it demands, has led us to open old boxes of photographs and papers. Yet this gesture is also a return to the past, a way of briefly restoring what is no longer there and easing, however provisionally, the emptiness of loss*.

*Do not let Mum read these -she said-. She will be sad*.

“*Hi Mum, how are you? I suppose, tired* … *As always, I am snoring when you arrive and cannot say hello or ask about your work, but that is what pen and paper are for. So… I think I watch too much silly TV, but I cannot help it, you would not let me, because, etc., etc., If I am still asleep when you get home, please carry me to bed and leave me a note if you want me to wake up*.

*Urgent. My sibling wanted to go with you; he was (a small drawing of a crying face and a flower) because he missed you.”*

*Inside one box, in a zip*-*lock bag, there were three letters like this, written in my daughter's handwriting more than 20 years ago, with drawings of moons, stars, and good nights. They spoke, without reproach, of a tired, absent mother who was non-etheless awaited unconditionally*.

*I remember that period clearly: multiple jobs without contracts, the feeling that my children were unprotected, and a desire that slipped away under the weight of everyday demands. A constant bodily tension persisted between the longing to continue studying, to have part of my degree from my country of origin recognized, and motherhood in a place that was not my own, without a network to hold things together and prevent them from becoming undone. Shortly after arriving here, I began the process of credential recognition at two universities, hoping that at least some of the courses I had completed would be accepted. For several months, I attended classes on my only day off from service work. It was unsustainable. My age, prior experience, and earlier education likely marked a distance between me and my classmates. Sometime later, on a train home, I sat next to someone my age reading a document from a* distance learning *university. When I got home, I looked it up. That institution eventually allowed me to complete my degree, my master's, and my doctorate*.

Many readers may recognize elements of this scene, although the meanings and consequences of such experiences are unevenly distributed. Rather than depicting an exceptional individual trajectory, the scene points to broader structural conditions shaped by intersecting inequalities related to gender, migration, age, care responsibilities, and academic precarity, which affect some academic trajectories more than others.

This article addresses the question: *how and why do migrant women experience delayed academic consolidation in European academia, and what are its implications for active aging?* Delayed academic consolidation is understood here as a prolonged, non-linear, and structurally conditioned process through which academic recognition, stability, and participation are deferred across the life course. Rather than reflecting individual shortcomings or atypical career decisions, it emerges from the cumulative effects of intersecting inequalities that shape access to opportunities, resources, recognition, and stable employment within academia.

The concept of delayed academic consolidation challenges dominant representations of academic careers as linear, meritocratic, and synchronized with normative life-course expectations (Savigny, 2019). To analyze this process, the article brings situated narratives into dialogue with three complementary theoretical perspectives. Feminist intersectional perspectives (Crenshaw, 1989; Collins and Bilge, 2020) provide a framework for examining how gender, migration, age, and care responsibilities interact to shape academic trajectories. Recognition theory (Fraser, 1995, 2000) provides a lens for analyzing how material inequalities, precarity, and symbolic misrecognition structure unequal conditions of participation within academia. Finally, the Active Aging framework (World Health Organization, 2002) situates these inequalities within a life-course perspective, highlighting their implications for health, participation, and security over time.

The empirical material was generated through iterative cycles of narrative writing and collective interpretation, resulting in a series of scenes that capture key moments of rupture, transition, and reconfiguration across the life trajectories of migrant women academics. Rather than treating these narratives as illustrative cases, the article approaches them as sites of situated theorization through which broader institutional dynamics can be interpreted and understood. In doing so, lived experiences are understood not merely as evidence of academic inequality, but as a mode of knowledge production that makes visible how academic careers are shaped by unequal regimes of recognition, participation, and stability.

By bringing feminist intersectional perspectives, recognition theory, and the Active Aging framework into dialogue, the article contributes to a counter-hegemonic understanding of academic careers as life-course processes shaped by cumulative inequalities. It shows how delayed academic consolidation extends beyond career progression to shape the conditions under which health, participation, and security are achieved over time, thereby linking academic trajectories to broader processes of aging, vulnerability, and social exclusion.

## Literature review

Recent literature has shown how the unequal distribution of power in European higher education, shaped by institutional dynamics of sexism, racism, and colonialism, continues to structure academic careers, particularly affecting racialized and migrant women (Savigny, 2019). Despite ongoing gender equality efforts, women remain underrepresented in senior academic positions and continue to face direct and indirect forms of discrimination throughout their careers (Verge Mestre, 2021; Winslow and Davis, 2016). These patterns are best understood through an intersectional lens, which highlights how multiple axes of inequality interact across institutional contexts (Crenshaw, 1989; Collins and Bilge, 2020). This perspective provides the first analytical pillar of the study by showing how gender, migration, age, and care responsibilities interact to shape differentiated academic trajectories.

At the same time, migration research points to a shift from the general feminization of migration toward the feminization of skilled migration, with highly educated women increasingly engaging in transnational mobility, often at higher rates than their male counterparts. This trend is particularly relevant within European higher education systems, which are structurally reliant on mobility and internationalization (Dumitru, 2017). However, as Morley et al. (2018) show, the institutional discourse of internationalization is frequently presented as ideologically neutral, while the actual mobility of migrant academics is shaped by hierarchies of class, ethnicity, gender, and geopolitical origin that determine who can move, under what conditions, and with what forms of recognition.

This resonates with Kim's (2010) concept of *transnational identity capital*, which describes how mobile academics may remain positioned as “strangers” within host institutions even after formal incorporation. This points to forms of symbolic misrecognition that formal credentialing alone cannot resolve. Yet, the post-migration academic trajectories of highly skilled migrant women academics remain underexplored, particularly in relation to long-term career consolidation, recognition, and wellbeing (Christou and Kofman, 2022).

Further research shows that gender inequalities in academia cannot be reduced to positional underrepresentation alone but must also be analyzed in relation to the temporality of academic careers. Women's trajectories are more likely to be marked by discontinuities, slower progression, and interruptions, reflecting the interaction between organizational logics, care responsibilities, and intersecting inequalities related to gender, migration, and age (Savigny, 2019). Biased evaluation practices, everyday micro-inequities, and expectations of uninterrupted availability remain fundamentally misaligned with the unequal distribution of care responsibilities, constraining women's ability to sustain continuous and competitive careers within meritocratic systems that obscure unequal starting conditions (Acton and Glasgow, 2015).

These inequalities are intensified by neoliberal transformations in higher education, characterized by performance regimes, audit cultures, and the devaluation of relational and emotional labor. Such conditions contribute to cumulative exhaustion and undermine the sustainability of academic careers over time, reflecting broader dynamics of time, energy, and life extraction under neoliberal academic regimes (Acton and Glasgow, 2015; Gokturk and Tulubas, 2021). Gill (2014) situates academic labor within critical cultural labor studies, documenting growing precarity, work intensification, and surveillance while challenging institutional portrayals of academic work as privileged and vocation driven.

These tensions have significant implications for wellbeing, retention, and career longevity, and can be analyzed through Fraser's (2000, 1995) distinction between redistribution and recognition. While precarious employment conditions limit access to material resources, career stability, and professional continuity, symbolic hierarchies simultaneously shape whose trajectories, experiences, and knowledge are considered legitimate and worthy of recognition within academia. From this perspective, delayed academic consolidation cannot be reduced to precarious employment or career instability alone. It is produced through the interaction of material inequalities and processes of misrecognition that shape access to resources, professional legitimacy, and opportunities for participation within academia. Fraser's framework therefore constitutes the second analytical pillar of this study, illuminating how inequalities of redistribution and recognition interact to shape migrant women academics' experiences of delayed academic consolidation.

Institutional responses, including the proliferation of gender equality policies across European universities, have sought to address these disparities. However, their transformative potential remains constrained by both explicit resistance and more diffuse mechanisms of symbolic compliance and institutional inertia. These dynamics reflect a deeper epistemological struggle, whereby feminist approaches continue to challenge an academic culture that marginalizes gender as a legitimate analytical and organizational concern (Verge Mestre, 2021). Although formal commitments to equality may expand recognition at the level of policy, they do not necessarily transform the institutional practices through which recognition is enacted. Ahmed (2021) analysis of institutional complaints illustrates how academic institutions may neutralize grievances by shifting attention from structural problems to those who report them. This organized silencing constitutes a form of institutional non-recognition that allows inequalities to persist despite formal commitments to inclusion and equality.

Adopting a life-course perspective provides a third analytical lens for understanding how academic inequalities accumulate and unfold over time. Research on migrant women shows how trajectories are shaped by the intersection of gender, mobility, and care responsibilities, resulting in patterns of high mobility, fragmented careers, and constrained recognition (Christou and Kofman, 2022). These cumulative processes are particularly significant when examined through the Active Aging framework (World Health Organization, 2002), whose dimensions of health, participation, and security provide a useful basis for analyzing the long-term consequences of academic inequalities. In this sense, academic careers can be understood not only as professional trajectories but also as pathways shaping conditions of social inclusion and vulnerability over time. For migrant women academics, delayed access to stable employment and institutional recognition may generate insecurities that affect future autonomy, material stability, health, and participation in later life.

Empirical studies illustrate how these dynamics are experienced in practice. Gokturk and Tulubas (2021) show how the intersection of patriarchal norms and neoliberal expectations compels women academics to extend working hours, defer personal life decisions, and internalize norms of constant productivity. Similarly, Crowley-Henry et al. (2025) demonstrate how the careers of migrant women scholars are shaped by inequalities associated with gender, migratory status, class, and caregiving responsibilities. Their findings challenge the notion of academia as a neutral meritocratic space by revealing persistent processes of othering and differential recognition embedded within institutional structures. These dynamics limit some academics' ability to participate on equal terms within institutional life (Fraser, 1995, 2000).

Complementary qualitative research highlights the centrality of emotional and relational labor in the everyday experiences of migrant academics. Navigating racialized and gendered institutional environments often requires sustained efforts of adaptation, visibility management, and overperformance (Tsouroufli, 2025). While these strategies may facilitate short-term inclusion, they also generate cumulative costs in terms of health, wellbeing, and belonging. Moreover, such forms of labor are frequently appropriated within institutional equality discourses, creating a paradox in which practices of resistance become absorbed by the very structures they seek to challenge.

Despite the growing body of research on gender inequality, migration, and academic precariousness, important gaps remain. Existing studies have largely focused on early- and mid-career stages or have treated gender and migration as separate analytical dimensions, paying less attention to how inequalities accumulate across the life course and shape future aging conditions (Christou and Kofman, 2022; Savigny, 2019; Verge Mestre, 2021). As a result, relatively little is known about how deferred recognition, instability, and exclusion become embedded within longer-term academic trajectories. In response, this article conceptualizes delayed academic consolidation as a prolonged, non-linear, and structurally conditioned process through which migrant women academics experience deferred recognition, participation, and stability as a consequence of cumulative inequalities across the life course.

By addressing these gaps, this article advances a feminist intersectional and life-course approach to academic careers, showing how structural inequalities become embodied and materialized over time. It brings feminist intersectional perspectives, recognition theory, and the Active Aging framework into dialogue to examine how delayed consolidation is produced and how it shapes migrant women academics' opportunities for health, participation, and security throughout the life course. Empirically, it contributes a collaborative autoethnographic account of migrant women academics, foregrounding situated and reflexive forms of knowledge production that are often absent from dominant accounts of academic work.

## Collaborative intersectional autoethnography

This study adopts a collaborative intersectional autoethnographic methodology (Adams and Herrmann, 2020; Humphreys, 2005; Spry, 2001; Suominen, 2006) to examine the academic trajectories of migrant-origin women within European higher education, understood as situated, unequal, and shaped across the life course. Autoethnography is particularly suited to this aim as it enables the analysis of lived experience while situating it within broader institutional and structural contexts (Ellingson and Ellis, 2008).

Rather than treating experience as merely illustrative, autoethnography is employed here as a critical and reflexive mode of inquiry in which embodied narratives function as analytical material. This approach challenges the separation between experience and theory by treating narrative production and interpretation as mutually constitutive processes. Knowledge is thus understood as situated, relational, and shaped by positionality, institutional location, and biographical trajectories. In line with feminist epistemologies, lived experience becomes a site through which structures of inequality and power relations can be interrogated. Intersectionality (Collins and Bilge, 2020; Crenshaw, 1989) provides a central analytical lens to examine how gender, migration, age, and care responsibilities interact relationally across the life course, producing differentiated academic trajectories. The focus on migrant women academics captures a specific intersectional configuration in which these dimensions converge, often resulting in non-linear and delayed forms of academic consolidation.

### Research team and positionality

The research team consists of four women academics aged between the ages of 43 and 56, all recently appointed as lecturers at a public European higher education institution. Three of the authors have migrant backgrounds, including two from Latin America and one from another European country. A fourth author developed a substantial part of her academic career in Latin America after leaving Spain due to the limited opportunities for academic employment that followed the 2008 financial crisis. These trajectories have informed the research team's sensitivity to issues of mobility, inequality, and structural barriers within higher education.

These differentiated yet interconnected trajectories shape both the production and interpretation of the empirical material. The autoethnographic scenes included in this article were produced by two of the migrant authors. Prior to the writing process, all four authors engaged in a series of collective discussions reflecting on their academic trajectories, experiences of migration, precarity, care responsibilities, and professional recognition. Through this exploratory dialogue, four recurrent experiential dimensions were identified inductively and subsequently used to guide narrative production:

1. Uprootedness and relational fragility;
2. Imposture;
3. Health and wellbeing; and
4. Modes of enacting the profession.

For each dimension, guiding prompts were developed to stimulate experiential writing and analytical reflection, focusing on significant episodes, embodied experiences, turning points, and perceived forms of inequality, recognition, or exclusion.

### Data production and analytical process

These analytical dimensions were iteratively refined through an ongoing collective process involving all four authors. Although the autoethnographic scenes were produced by two of the migrant authors, all four authors participated in the collective reading, discussion, interpretation, and refinement of the analytical categories. The analysis therefore drew on the diverse positionalities of the entire research team, including migrant and non-migrant positionalities, generating a shared and dialogical interpretation of the narratives.

The process followed iterative cycles of narrative writing, collective reading, and joint interpretation. In each cycle, experiential accounts were produced in response to guiding questions, followed by dialogical discussion in which convergences, tensions, and divergences were identified and collectively analyzed. Rather than being treated as noise, moments of disagreement were approached as analytically productive, enabling the refinement of categories and the deepening of interpretation. Through repeated dialogue, the authors identified recurring patterns across narratives, contrasted individual experiences, and related these patterns to broader theoretical concepts derived from feminist intersectional perspectives, recognition theory, and the Active Aging framework.

This process aligns with Ellingson and Ellis (2008) notion of co-constructed meaning, extending autoethnographic analysis beyond individual reflection toward collective epistemic production. The method is further characterized by a continuous movement between empirical material and theoretical frameworks, where scenes are treated as analytically significant moments in which structural tensions, contradictions, and inequalities become visible.

The collaborative interpretive process enabled the identification and refinement of delayed academic consolidation as an emergent analytical construct capturing the cumulative, non-linear, and structurally conditioned character of the authors' academic trajectories. In this sense, the scenes function as counter-hegemonic narrative units that render visible forms of misrecognition, precarity, and inequality often obscured in dominant academic discourses.

### Rigor and quality criteria

Methodological rigor is ensured through transparency of process, explicit articulation of analytical decisions, sustained reflexivity, coherence between narrative material and interpretation, and iterative collective discussion among the authors. Rather than relying on replicability, rigor is defined in terms of credibility, reflexive depth, interpretive consistency, and transparency regarding the co-construction of meaning.

Following narrative and autoethnographic scholarship (Conle, 1999; Connelly and Clandinin, 1995), the study prioritizes resonance over representativeness, understood as the capacity of narratives to generate recognition, analytical insight, and shared understanding through sustained interpretive engagement.

### Ethical considerations

The study is grounded in principles of relational ethics, emphasizing care, reflexivity, responsibility, and ongoing attention to the positionalities of the authors in the production and interpretation of personal narratives. All accounts refer exclusively to the authors' own experiences and avoid identifiable third parties.

Beyond its analytical aims, the study also seeks to contribute to critical reflection on more just, sustainable, and livable ways of inhabiting academic life.

## Autoethnographical scenes

### Uprootedness and relational fragility

When and how do experiences of rootlessness and relational fragility emerge in academic trajectories?

***Scene 2 (Author B): starting over, again and again***

*The first time I became aware of a sense of uprootedness and fragility was when I left my country to study abroad. Until then, studying had represented a path of personal discovery and growth, but arriving in a country so different, culturally, and socially, profoundly transformed my perception of myself. There, for the first time, I became aware of my identity as something visible, marked in my body, in the way I communicated and related to others. I felt my difference from “others” in a direct, almost physical way. Although the university experience was stimulating, over time I began to feel that something of myself was disappearing. Experiences of discrimination and marginalization involving foreign colleagues and professors revealed deep exclusionary structures, while also rendering me increasingly vulnerable*.

*It was here that this fragility became more explicit. Language barriers, the lack of networks and administrative constraints made me feel alone and unprotected. When I confronted situations of discrimination based on my origin and gender, reprisals followed that curtailed my continuity within academia and led to a prolonged period of precariousness and a new migration. Each transition required rebuilding relationships, adapting once again, and repeatedly justifying my presence, my competence and my trajectory. Uprootedness and relational fragility, in varying intensities, have remained tied to my condition as a foreign woman, an adult, and a mother*.

*Now in my fifties, one would expect an academic trajectory to be consolidated. Yet even today, expressing my age and employment status makes me feel exposed, vulnerable, and misaligned with what is expected of an academic career. This is not merely a matter of identity or personal perception; it reflects persistent forms of symbolic and material subordination*.

Scene 2 illustrates how uprootedness emerges not as a singular effect of international mobility, but as a cumulative process of temporal, relational, and institutional disruption across the academic life course. Mobility becomes the initial condition through which a longer-term pattern of precarization unfolds, affecting the stability of social ties, institutional belonging, and professional continuity. Rather than being reducible to geographical displacement, uprootedness operates as a form of temporal and relational instability produced by repeated institutional transitions, precarious employment, and the need to continuously rebuild academic, social, and institutional attachments.

The scene also introduces a preliminary life-course dimension by revealing the misalignment between chronological age and academic consolidation. This misalignment signals the need to understand academic trajectories not as linear progressions, but as uneven temporalities shaped by structural instability. From this perspective, the Active Aging framework (World Health Organization, 2002) becomes relevant for conceptualizing how early- and mid-career academic instability may shape later-life conditions of participation, security, and wellbeing.

In this sense, uprootedness and relational fragility are not merely consequences of migration. They are interconnected processes through which delayed academic consolidation is experienced, embodied, and reproduced across the life course.

### Imposture

Why do feelings of imposture emerge, and how are they experienced?

***Scene 3 (Author*** ***A): you are never where you are supposed to be***

*Academia is one of the institutions that strongly reflects and reproduces patriarchal, conservative, classed and racialized social orders and, as such, demands from some bodies more than from others the adoption of forms of imposture, often as a mode of subsistence or* self-defense *within an environment governed by the preservation and reproduction of singular, hegemonic and self-referential knowledge*.

*In my case, I do not embody a dissident body; however, the experience of sustained precariousness, combined with migrant motherhood, always places you elsewhere in relation to others, not only materially, but as a lived condition. The vulnerability to which precariousness exposes you, when you have children in your care and have migrated alone, is difficult to understand for those who have not lived it. The demands of everyday survival prevented me from dedicating time to my education and profession. I felt compelled to compensate for a discontinuous and complex trajectory when compared to others whose paths were far more linear*.

*It then seems that you are never where you are supposed to be, a feeling closely linked to imposture. The sense is that you never quite arrive; by being everywhere, you are nowhere. What sustained me was not losing sight of my interests and desires, even when they were often distant from what is expected in academia. The constant pressure to accumulate quantifiable “merits” and to meet an unending sequence of evaluations turns academia into an increasingly unreachable and, at times, inhospitable space. Its productivist logic expels everything that does not have an evident utility in neoliberal terms. In this way, imposture is imposed upon us: a way of being misaligned that compels us to constantly ask ourselves what we are doing here. Feminist thought, by contrast, has provided a language to name that what tensions us both physically and symbolically, for instance through the highly compelling notion of the capital-life conflict, which opens difficult questions and invites us to cultivate a sensitivity for finding alliances in unexpected places*.

***Scene 4 (Author B): performing a role that is not mine***

*I have experienced imposture particularly when I have had to change research topics due to precariousness and the temporary nature of my employment, without being able to develop any of them in a sustained way. In some ways, this has enriched my perspective on science, helping me recognize the need for a holistic and interdisciplinary approach to address the complexity of contemporary issues. However, it has also hindered my professional trajectory, as I have not been able to develop or present a clearly defined research line. I have often found myself in a kind of limbo between interdisciplinarity and coherence, which has increased my sense of professional insecurity*.

*At the same time, I* frequently *find myself producing for the sake of producing, without the time to reflect on or truly integrate that knowledge. Tasks become automatic and mechanical, leaving little room for deeper engagement. It becomes difficult to prepare work in a substantive way, and I frequently end up improvising-a strategy that is, unfortunately, all too common in academic practice. I also feel like an impostor occupying an academic position that is typically associated with a much younger age group*, regularly *no older than thirty, while remaining caught in the cycle of productivity and the constant need to prove my worth. I no longer have the energy or motivation to perform a role that does not correspond to me, either in terms of age or accumulated experience. This dissonance between the academic position I occupy, and my stage of life intensifies that sense of imposture, as if I were performing a role that the institution itself reserves for a different profile and life trajectory*.

Scenes 3 and 4 show how imposture emerges as a structured effect of recognition regimes within contemporary academia. Rather than reflecting an individual psychological disposition, imposture reveals the conditions under which academic legitimacy is continuously negotiated, questioned, and deferred.

The experience of “never being where one is supposed to be” captures a form of symbolic displacement produced through meritocratic and productivist logics that privilege linear, uninterrupted, and age-normative trajectories. Within this configuration, recognition is not secured through achievement alone but must be constantly performed, demonstrated, and revalidated.

From the perspective of Fraser's theory of redistribution and recognition (1995, 2000), these dynamics reflect a dual structure of injustice. First, material precarity limits sustained academic production, career continuity, and access to resources. Second, symbolic hierarchies devalue non-linear, migrant, and care-embedded trajectories, producing systematic misrecognition. Together, these processes undermine parity of participation by positioning certain academic subjects as permanently provisional.

An intersectional perspective (Crenshaw, 1989; Collins and Bilge, 2020) further illuminates how migration, motherhood, and age do not operate independently but mutually constitute one another, producing a persistent misalignment between embodied life trajectories and institutional expectations of academic progression. In this context, age functions not as a neutral biographical marker but as an evaluative category through which competence, potential, and academic legitimacy are implicitly assessed.

Imposture thus emerges as a socially produced condition rooted in institutional dynamics of recognition and exclusion. It is generated by academic structures and internalized as a mode of self-assessment under conditions of structural misrecognition. Ahmed (2021) work further shows how those who do not conform to dominant institutional norms are often repositioned as the problem, rather than the institutional arrangements that marginalize them. From this perspective, feelings of imposture are not simply the consequence of individual self-doubt or non-normative career trajectories. Rather, they reflect institutional processes that defer recognition while constraining individuals' capacity to claim legitimacy. As such, imposture can be understood as one of the subjective manifestations of delayed academic consolidation.

### Health and wellbeing

What are the implications of academic working conditions for health and wellbeing?

**Scene** ***5 (Author B): a continuous feeling of being overwhelmed***

*The professional conditions in which I am immersed shape my wellbeing and health. It is not simply a matter of workload or occasional stress; it is a continuous feeling of being overwhelmed, of not having a real space to breathe. Each day becomes an accumulation of tasks in teaching, management, research, and knowledge transfer that are difficult to reconcile with a human pace. I feel as though I am caught in a self-reinforcing cycle: just as I finish one task, another urgent demand emerges-another email, another meeting, another deadline* … Work *seems to occupy not only my day, but also my mind, body, and emotional state. At times, there is no mental space left for me. This leaves persistent fatigue, a kind of fog that settles in and is hard to dispel*.

*Bureaucracy and administrative work occupy an increasing part of my professional life. Email, which accumulates from early in the morning, has become an almost automatic trigger of anxiety. Teaching, which could be a creative and dynamic space, is often absorbed into this machinery. The more I do, the more is expected, and this continuous escalation takes its toll. It is deeply exhausting. Another element that intensifies this strain is the limited space for human interaction. Opportunities for connection are scarce within this accelerated work rhythm. With students, interaction is largely confined to class time; with colleagues, to formal meetings. Moments of shared time beyond task-oriented exchanges are almost non-existent. This gradually erodes relationships, the sense of belonging, and the possibility of mutual support. At times, I realize that weeks can pass without a single unhurried conversation within my professional environment*.

*These conditions extend beyond individual experience and have a clear impact on health and wellbeing. I have experienced periods of insomnia, anxiety, insecurity, and accumulated stress, as well as a level of mental and physical exhaustion that is difficult to ignore. Over time, I become aware that my overall satisfaction with life decreases, that it becomes harder to enjoy, to connect, to pause. It is as if work gradually occupies spaces that were once reserved for life itself. Recognizing this is painful, but it is also the first step toward seeking other ways of inhabiting the profession without allowing it to consume me from within*.

***Scene 6 (Author A): if Sisyphus were a woman today..***.

*It is not only a matter of physical exhaustion, but of a deeper form of fatigue: that of an overloaded mind. At times, I think that if Sisyphus were a woman today, she would not be represented as a strong, muscular male body, but rather as any one of us, carrying out the absurd and repetitive labor of endless return. If we update this metaphor and consider the burden of domestic work and unpaid care, this becomes quite evident. This largely invisible labor, if not performed by us, is often displaced onto other women*—*frequently migrants working in more vulnerable conditions. Yet, in our profession, this logic does not disappear; rather, it is added to what we already carry as a social mandate. In this sense, the organization of academic work does not stand apart from broader gendered inequalities but is entangled with them. Expectations and self-demands* regularly *seem to emerge from an abstract or diffuse place, if not from us, as we assume responsibility for responding to multiple and competing demands*.

*Being over 50, having migrated, and having experienced prolonged periods of precariousness makes it difficult to disengage from recurring episodes of anxiety and the fear of being left out at this stage of life. This inevitably erodes the capacity for enjoyment and disconnection. If the reproduction of life has historically depended on this alienating labor socially assigned to us, and if this logic is sustained through forms of inequality and the extraction of our time for life, it becomes essential to open up spaces where a life worth living is possible, not only for some, but for all*.

Scenes 5 and 6 show how academic working conditions produce sustained forms of embodied exhaustion that exceed episodic stress. Distress emerges as a structural condition of continuous work intensification, where academic labor extends into cognitive, emotional, and bodily domains.

Scene 5 illustrates how institutional demands generate a regime of permanent task accumulation in which work is never fully completed but continuously renewed. This produces a chronic state of overload characterized by fatigue, reduced recovery time, and the erosion of relational spaces within the workplace. Berg and Seeber (2016) concept of the *slow professor* helps interpret this overload as part of a broader culture of speed in academia, where corporatization, efficiency, accountability and standardization intensify time poverty and academic stress. Their argument shifts the focus from individual incapacity or poor time management to the structural organization of academic work. In this context, the absence of time for reflection, collegial connection, and recovery is normalized as a routine feature of academic life rather than recognized as a consequence of institutional conditions.

Scene 6 extends this analysis by situating exhaustion within the intersection of academic labor and social reproduction. The metaphor of Sisyphus captures the repetitive and seemingly endless character of academic and care work, where each completed task is immediately replaced by a new demand. This dynamic resonates with Bertuzzi (2026) concept of *emergenciocracy*, which describes how emergency logics become normalized as structural modes of governance. Applied to academia, this perspective helps explain how urgency, deadlines, and permanent responsiveness intensify work while rendering exhaustion an ordinary condition of professional life.

The gendered dimension of care work further reveals how invisible labor is unevenly distributed across bodies, often intensifying the burden carried by women in academia (Acton and Glasgow, 2015; Gokturk and Tulubas, 2021). Exhaustion therefore becomes cumulative, affective, and socially structured rather than merely individual.

Riach (2025) conceptualization of organizational aging helps interpret these scenes as more than accounts of work overload. They show how academic institutions organize aging through expectations of continuous productivity, bodily availability, and self-management. From this perspective, the exhausted academic body is not incidental to delayed consolidation; it is part of a temporal regime in which recognition remains tied to the capacity to keep producing despite accumulated strain.

Within this configuration, the Active Aging framework (World Health Organization, 2002) highlights how health, participation, and security are co-produced across the life course under conditions of structural constraint. Rather than assuming that aging naturally brings greater stability, the scenes show how sustained precarity undermines the foundations of active aging. Delayed academic consolidation therefore affects not only career progression but also future opportunities for health, participation, and security across the life course.

### Modes of enacting the profession

How are academic practices shaped and transformed over time? How are they experienced?

***Scene 7 (Author B): dignifying ourselves in academia***

*I believe this is the first time in many years that I have allowed myself to reflect on how I would like to inhabit academia. For a long time, I have operated almost on autopilot, responding to what was expected of me at each stage. It is difficult not to become absorbed by the logic of competition and productivity, as stability and recognition depend on it. This logic has become so normalized that expressions such as “we have all been through this” leave a profound sense of lack of protection, as if distress were an inherent and inevitable part of academic work. Coming from a different cultural background adds another layer of exposure: students who point out that “you still do not fully master Catalan” while navigating between several languages on a daily basis, or who perceive you as “too distant,” unaware that there is often little space to attend to one's own needs and emotions. At times, even colleagues have suggested difficulties of cultural adaptation*.

***Scene 8 (Author A): resisting the logic of exhaustion***

*Reflecting on my life trajectory helps to put certain insecurities into perspective. While working as an adjunct lecturer, I shared with students the working conditions and demands we face, which at times opened spaces for dialogue and a degree of horizontality. Over time, I feel less indebted to prescribed contents, which allows me to introduce my own interests and approach teaching from different entry points*.

*At the same time, academic space has gradually shifted from a place of reflection and learning to one of generalized management: of tasks, of ourselves, of our emotions, and of our academic trajectories. This has been a significant source of disillusionment, particularly the everyday realization that the extraction of time and data, from ourselves and from others, often results in limited or merely formal returns to society. Productivist demands push us to move continuously between fragmented teaching and research topics, shaped by changing teaching assignments, short-term funding, and weakly connected projects. In this context, this study itself becomes a space for awareness and repositioning. Engaging in both individual and collective reflection thus constitutes a commitment and a form of resistance to the imposed logic of exhaustion*.

Scenes 7 and 8 reveal how academic practice is enacted through an ongoing negotiation between institutional demands, professional values, and embodied constraints. Rather than appearing as a stable professional identity, enacting the academic profession emerges as a situated and evolving practice shaped by evaluative regimes, institutional expectations, and structural precarization.

Scene 7 highlights how continuous adaptation undermines coherence in academic practice, often experienced as operating on “autopilot.” The normalization of productivist expectations individualizes distress and obscures its structural origins, while everyday interactions reveal subtle forms of misrecognition linked to language, migration, and cultural positioning. These dynamics resonate with research on migrant academics' symbolic positioning within host institutions, where formal incorporation does not necessarily translate into full recognition or belonging (Kim, 2010).

Scene 8 introduces a partial reorientation of academic practice, in which pedagogical autonomy and reflexive awareness create limited spaces for resistance. However, these possibilities remain constrained by fragmented institutional structures and intensified evaluation regimes that reorganize academic work as continuous self-management. This resonates with Berg and Seeber (2016) critique of the accelerated university, which argues that reclaiming time for reflection, dialogue, and pedagogical care can operate as a form of resistance to the corporatized culture of speed, efficiency, and accountability in academia.

Across both scenes, an intersectional configuration of gender, migration, age, and language shapes differentiated conditions of academic participation and recognition. These dimensions operate relationally rather than independently, influencing how academic subjectivities are positioned within institutional hierarchies. From Fraser's perspective (1995, 2000), these dynamics can be understood as struggles over parity of participation, since unequal recognition and precarious working conditions shape who can participate in academic life on equal terms.

Taken together, these dynamics indicate that enacting the profession is not a linear process of professional development, but a continuous negotiation between the pursuit of recognition, the attempt to maintain professional continuity, and the exhaustion generated by prolonged precariousness and institutional productivity demands. Under contemporary neoliberal conditions, academic subjectivity is increasingly shaped by productivity, flexibility, and self-management (Gill, 2014). The negotiation of academic practice therefore becomes one of the ways in which delayed academic consolidation is experienced, contested, and re-signified across the life course.

## Discussion

This article has examined how migrant women academics experience delayed academic consolidation as a structurally produced and temporally embedded phenomenon, and how this process has implications for active aging. Across the four analytical dimensions, academic trajectories emerge not as linear careers but as uneven life-course processes shaped by intersecting regimes of recognition, precarity, and temporal constraints.

The analysis demonstrates that academic institutions operate through implicit norms of linear progression, uninterrupted productivity, and age-aligned consolidation, consistent with previous research on gendered and neoliberal academic cultures (Acton and Glasgow, 2015; Gokturk and Tulubas, 2021; Morley et al., 2018; Savigny, 2019). These norms structure conditions of recognition, positioning non-linear, migrant, and care-embedded trajectories as persistently provisional. This echoes previous work showing how migrant academics may remain positioned as institutional “strangers” despite formal incorporation (Kim, 2010). Within this framework, recognition is not merely symbolic but structurally mediated, shaping access to legitimacy, participation, and institutional continuity.

An intersectional perspective highlights how gender, migration, age, and care responsibilities operate relationally to structure academic subjectivities, echoing findings on migrant women academics' career and mobility trajectories (Christou and Kofman, 2022; Crowley-Henry et al., 2025). These dimensions cannot be understood as additive factors but as mutually constitutive processes that shape differentiated conditions of academic belonging, recognition, and participation. From the perspective of redistribution and recognition (Fraser, 1995, 2000), these intersectional inequalities become embedded in academic careers through both material and symbolic mechanisms. Material precarity limits access to resources, stability, and professional continuity, while symbolic misrecognition devalues non-linear, migrant, and care-embedded trajectories. Together, these processes undermine parity of participation by systematically disadvantaging trajectories that deviate from dominant academic norms and by delaying access to recognition, stability, and institutional legitimacy.

Postcolonial feminist scholarship further illuminates how these regimes of recognition are embedded within broader global hierarchies of knowledge production. Mirza (2009, 2018) work on race, gender, and higher education shows that racialized and migrant women academics may occupy positions of conditional belonging, where institutional inclusion does not necessarily translate into epistemic recognition, authority, or equal participation. This perspective reinforces the argument that delayed academic consolidation is shaped not only by national institutional arrangements but also by wider postcolonial structures of academic recognition and knowledge production.

The Active Aging framework (World Health Organization, 2002) allows the consequences of delayed academic consolidation to be situated within a life-course perspective. The findings show that prolonged precarization, institutional instability, and deferred recognition accumulate over time, shaping differentiated possibilities for health, participation, and security. This interpretation extends previous research showing that migrant women's academic trajectories are frequently marked by mobility, fragmented careers, and constrained recognition (Christou and Kofman, 2022). In line with Riach (2025) understanding of organizational aging as institutionally produced, the analysis shows how academic institutions attach value to particular temporalities of career progression. Linear, uninterrupted, and “on-time” trajectories are more easily recognized as signs of academic potential and legitimacy, whereas migrant, care-embedded, and precarious trajectories are more likely to be rendered delayed, provisional, or insufficiently consolidated.

Taken together, these findings suggest that delayed academic consolidation should not be understood as an individual deviation from normative career paths. Rather, it should be conceptualized as a structurally produced life-course process through which intersecting inequalities accumulate over time. Importantly, delayed academic consolidation is analytically distinct from academic precarity or non-linear careers alone. While these concepts capture instability, discontinuity, or interrupted progression, delayed academic consolidation highlights the cumulative deferral of recognition, participation, and stability across the life course. In this respect, the findings resonate with previous research documenting how academic careers are shaped by discontinuities, unequal recognition, and gendered life-course constraints (Crowley-Henry et al., 2025; Savigny, 2019).

The article therefore contributes to rethinking academic careers as life-course processes shaped by intersecting inequalities rather than as neutral, liner, or purely meritocratic trajectories. By bringing feminist intersectional perspectives, recognition theory, and the Active Aging framework into dialogue, the study shows how structural inequalities become embodied and materialized over time through delayed academic consolidation. In this sense, delayed academic consolidation operates as a mediating life-course process through which gendered, migratory, age-related, care-related, material, and symbolic inequalities shape future opportunities for wellbeing, participation, and security.

Consistent with scholarship on neoliberal academic environments, work intensification, and gendered and migrant precarity (Gill, 2014; Gokturk and Tulubas, 2021; Tsouroufli, 2025), the findings demonstrate that these inequalities affect not only professional advancement but also long-term opportunities for wellbeing, participation, and security. Berg and Seeber (2016) critique of the accelerated and corporatized university further reinforces the need to interpret academic stress, time poverty, and exhaustion as structural conditions rather than individual failures of adaptation. Delayed academic consolidation should therefore be understood not only as a matter of career progression but as a life-course process that shapes the conditions under which sustainable academic participation and active aging become possible or are progressively undermined.

## Conclusions

***Scene 9 (Author C): collectivizing distress***

“*Lying in bed, after 3 h of collective discussion devoted to reading and resonating with the scenes, a phrase comes to me: a Sisyphus stone that, when carried together, weighs less… Today, we have collectivized a shared distress that, until now, we had not had the opportunity to name and hold together within a cared-for academic space.”*

The final scene marks not only an affective closure but also an epistemic reconfiguration of the research process itself. Through collective reading and interpretation, distress is displaced from an individualized register into a shared, socially produced, and structurally intelligible condition. In this movement, the collaborative autoethnographic process becomes both method and object of analysis: a site where experience is not simply narrated but actively transformed into critical knowledge about academic structures.

Rather than treating lived experience as illustrative material, the collective engagement with the scenes produces a form of situated theorization in which meaning emerges through relational interpretation. This methodological gesture is central to the article's contribution: it shows how collaborative autoethnography can operate as a mechanism of epistemic translation, where affective experiences of exhaustion, misrecognition and vulnerability are reassembled as evidence of institutional arrangements governing academic life.

### Across the interpretative process, several cross-cutting analytical insights emerged:

#### Narrating vulnerability as epistemic production

Narrating from vulnerable positions functions as a situated form of knowledge production that makes visible the embodied and relational dimensions of academic labor. At the same time, it exposes the tensions inherent in academic writing itself, where demands for abstraction, coherence, and detachment must be continuously negotiated alongside ethical concerns around exposure, care, language and self-representation.

#### Interrupted academic agency and life-course constraint

Precarious employment and continuous evaluation regimes restrict not only professional stability but also biographical agency. Academic trajectories are therefore not simply individual pathways, but structurally conditioned processes shaped by migration histories, care responsibilities and life-course positioning, which delimit the capacity to actively shape one's academic becoming. These dynamics contribute to processes of delayed academic consolidation by limiting access to stable academic positions, institutional recognition, and opportunities for long-term career development.

#### Re-situating trajectories in historical time

Individual trajectories are inseparable from broader socio-economic transformations, including the 2008 financial crisis, austerity policies, and the COVID-19 pandemic. These events operate as structural accelerators of precarity and institutional instability, reinforcing the importance of reading academic careers as historically produced rather than individually authored.

#### From deficit narratives to situated forms of agency

While the scenes foreground vulnerability and exhaustion, collective interpretation reveals forms of situated meaning-making under constraint. This reconfiguration does not soften structural critique but rather specifies how academic life is sustained in practice under conditions of limited recognition and resources.

#### Intersectionality as relational structuring

Intersectionality operates here not as a descriptive taxonomy but as an analytical framework for understanding how gender, migration, age, care responsibilities, language and precarity co-constitute academic positioning. These dimensions function relationally, producing differentiated experiences even within apparently similar institutional locations. Rather than representing separate sources of disadvantage, they interact through institutional norms, linguistic expectations, and evaluative regimes that shape unequal opportunities for recognition, participation, and academic continuity.

#### Care, relationality and everyday political practices

The collective reading also highlights the political significance of everyday practices of care, listening, boundary-setting, and shared language-making. Rather than exceptional interventions, these emerge as ordinary yet essential practices through which academic and personal life are sustained under conditions of pressure and temporal scarcity. In this context, Segato (2021) work resonates as an invitation to reclaim the political significance of practices historically associated with feminized domains, such as care, relationality and listening, and to recognize their role in sustaining more livable academic trajectories while opening space for alternative political languages. These practices do not simply mitigate distress; they create conditions for naming harm, collectivizing vulnerability, and imagining academic life otherwise.

#### Toward livable academic trajectories

Taken together, the analysis suggests that sustaining academic life under conditions of structural precarity requires more than individual strategies of adaptation or resilience. It demands attention to how institutions organize time, recognition, participation, and care across the life course.

From an Active Aging perspective (World Health Organization, 2002), health, participation and security are shaped by academic trajectories over time. Precarity, evaluative pressure and misrecognition accumulate across the life course, constraining long-term sustainability, autonomy, and wellbeing within academia. The findings suggest that delayed academic consolidation may restrict opportunities for active aging by reducing access to stable employment, limiting meaningful participation, and generating cumulative effects on physical and emotional wellbeing, security, and participation.

In parallel, Fraser's theory of recognition (1995, 2000) enables us to understand distress not as an individual psychological condition but as the effect of structurally unequal regimes of participation. Parity of participation is systematically undermined when institutional norms privilege linear, continuous and early-consolidated academic biographies, while migrant, care-embedded, linguistically marked and non-linear trajectories are rendered delayed, provisional or insufficiently legitimate.

In sum, the article contributes both methodologically and conceptually. Methodologically, it demonstrates the epistemic and political potential of collaborative autoethnography as a mode of inquiry that transforms distress into structural insight. The collective process does not merely document lived experience but actively reconfigures it, making visible how affect, biography and institutional structures are co-constituted within academic life.

Conceptually, the study advances the notion of delayed academic consolidation as a distinct analytical framework for understanding how intersecting inequalities shape academic trajectories across the life course. Unlike approaches focused solely on precarity or non-linear careers, delayed academic consolidation captures the cumulative effects of deferred recognition, participation and stability, as well as their implications for future aging conditions.

Ultimately, the study shows that delayed academic consolidation is not an individual deviation from normative trajectories, but a structurally produced life-course outcome shaped by intersecting inequalities in recognition, participation and access to stability. By bringing feminist intersectional perspectives, recognition theory and the Active Aging framework into dialogue, the article demonstrates how migration, gender, age, care responsibilities, language and precarity become embedded in academic careers, shaping long-term opportunities for recognition, participation, wellbeing and security across the life course.

Making academic trajectories livable therefore requires not only individual resilience, but a transformation of the institutional conditions that govern recognition, participation, care and the organization of academic time, enabling more equitable opportunities for wellbeing, security and active aging across the life course.

## Data Availability

The raw data supporting the conclusions of this article will be made available by the authors, without undue reservation.
